# Molecular hallmarks of adult T cell leukemia: miRNA, epigenetics, and emerging signaling abnormalities

**DOI:** 10.1186/1742-4690-11-S1-O54

**Published:** 2014-01-07

**Authors:** Makoto Yamagishi, Dai Fujikawa, Naoya Kurokawa, Ai Soejima, Ryutaro Takahashi, Naoki Sakai, Shota Nakagawa, Kazumi Nakano, Seiichiro Kobayashi, Atae Utsunomiya, Kazunari Yamaguchi, Kaoru Uchimaru, Seishi Ogawa, Toshiki Watanabe

**Affiliations:** 1 Graduate School of Frontier Sciences, The University of Tokyo, Tokyo, Japan; 2 Institute of Medical Science, The University of Tokyo, Tokyo, Japan; 3 Department of Hematology, Imamura Bun-in Hospital, Kagoshima, Japan; 4 Department of Safety Research on Blood and Biologics, National Institute of Infectious Disease, Tokyo, Japan; 5 Faculty of Medicine, The University of Tokyo, Tokyo, Japan

## 

The molecular hallmarks of ATL comprise outstanding deregulations of signaling pathways that control cell cycle, apoptosis resistance, and proliferation of leukemic cells. Using integrative analyses of primary ATL cells, we discovered unique molecular characteristics of ATL (Yamagishi et al., Cancer Cell, 2012). Genetic and epigenetic disruption leads to numerous gene expression alterations that dominate disorders of homeostasis and characteristics of ATL. In particular, a novel tumor suppressor miR-31 is completely lost in all ATL cases, leading to constitutive NF-kB activation via NIK overexpression. Polycomb family directly involves in the silencing of miR-31, providing a novel interconnection between epigenetic reprogramming and NF-kB pathway. In addition, we recently unveiled molecular mechanisms how Polycomb-dependent epigenetic perturbation is abnormally sustained in HTLV-1 infected and leukemic cells. We discuss the recent discovery of molecular hallmarks of potential generality, an abnormal miRNA pattern and epigenetic reprogramming, which strongly involve the imbalance of the molecular network of lymphocytes. Because epigenetic marks are potentially reversible, development of genuine epigenetic-targeted therapy drugs holds great promise in HTLV-1-related diseases. Furthermore, we also introduce additional signaling pathways affecting leukemic cell fate. Pathway analysis based on our comprehensive dataset and biological studies suggest breaking down of the essential signaling pathways such as Hedgehog and p38 pathways, which support the biological properties of ATL. Because these organized principles may be directly associated with the clinical traits of ATL, targeting the one outstanding hallmark or co-targeting of multiple molecular hallmarks in mechanism-guided combinations will result in more effective and durable therapies for aggressive ATL.

